# Supplementary material for: Altered Tau Kinase Activity in rTg4510 Mice after a Single Interfaced CHIMERA Traumatic Brain Injury
Source: Int J Mol Sci. 2023 May 29;24(11):9439. doi: 10.3390/ijms24119439 (PMC10253362; doi:10.3390/ijms24119439)
Supplement: Supplementary file 1 [file ijms-24-09439-s001.zip › ijms-2416588-SM.pdf]

A

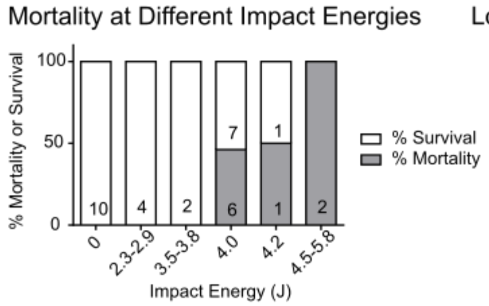

B

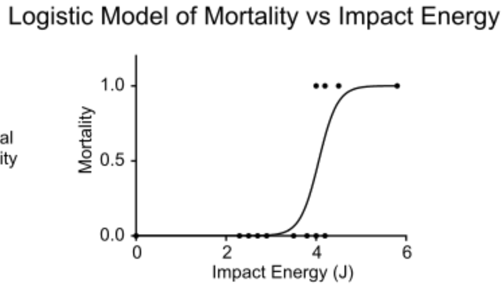

C

Causes of Death or Humane Endpoint

Cause	N
Death immediately after TBI, without external bleeding or visible intracranial hematoma upon dissection	2
Death immediately after TBI, with bleeding from eye/nose, or visible skull fracture/intracranial hematoma upon dissection	4
Humane endpoint reached at minutes to 6hr after TBI, due to irresponsiveness/head tilt, seizure-like behavior, or very laboured breathing	3

D

Tau antibody	Antigen/Recognition target	Source
CP13	human tau pSer202	P Davies
PHF1	human tau pSer396/Ser404	P Davies
MC1	human tau in (early) pathological conformation: exon10, aa 5-15 & 312-322	P Davies
DA9	total tau aa 102-140 (human/mouse)	P Davies
AT8	human pSer202/Thr205 (pSer199); early tangles	Thermo MN1020B

Figure S1. Mortality of 4-mo rTg4510 at different impact energies. (A) Breakdown analysis showing % mortality and % survival at different impact energy ranges. Numbers on the bar represent the number of mice that died/survived from the impact energy range. Note that the 7 mice that survived 4.0J impact and the 1 mouse that survived 4.2J impact were combined into one TBI group in this study. (B) Logistic regression model of mortality as a function of impact energy. The model is best fitted with the equation $y = 1/(1+e^{-4.831(x-4.060)})$, where y=1 indicates total mortality and y=0 indicates total survival. R^2=0.4098 (C) Summary of cause of death of animals that did not survive the TBI procedure.

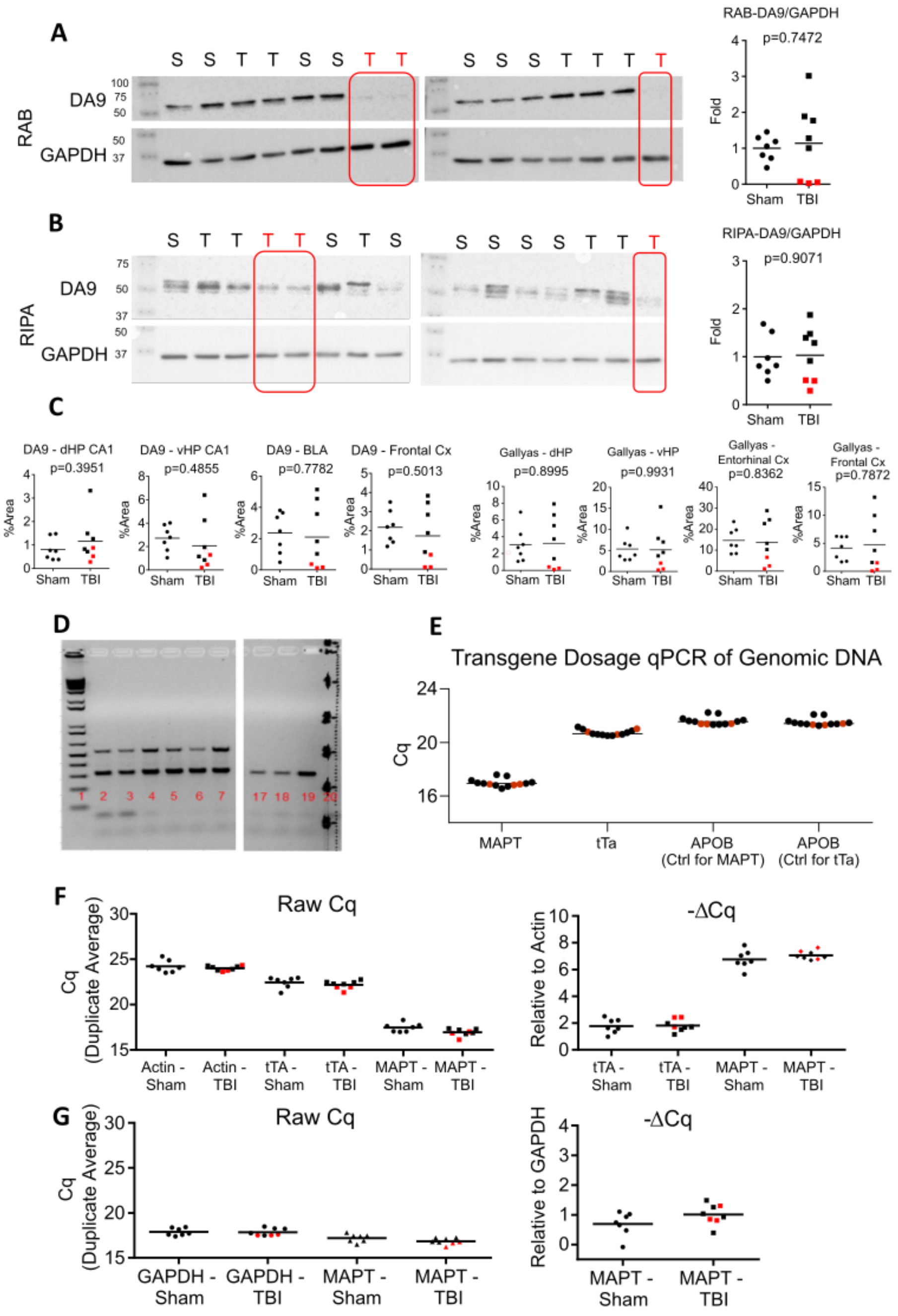

Figure S2. Western blot and genotype analysis of rTg4510 mice. (A-B) Western blotting and quantification of total tau in (A) RAB and (B) RIPA fractions of brain homogenates using DA9. All sham and TBI samples are shown. Three TBI samples (highlighted in red) showed unexpectedly low total tau levels, even much lower than sham. The results are consistent in both RAB and RIPA fractions. T-tests were used for all analyses. (C) Replicates of plots from Fig3A and Fig3B are shown, with the 3 TBI samples showing unexpectedly low tau level from (A) and (B) highlighted in red. (D-G) TBI mice with high and low levels of tau did not differ in genotyping, transgene dosage, or transgene expression. (D) Genotyping reconfirmation of rTg4510 mice. Lanes: 1: DNA ladder. 2: Sham rTg4510. 3 and 4: TBI mice with regular level of total tau. 5-7: TBI mice with low tau levels. 17-19: Non-transgenic mice. (E) Transgene dosage was performed using qPCR and confirmed a similar transgene copy number among the entire rTg4510 cohort, using 50 ng of genomic DNA as input. MAPT: tau transgene; tTA (tetracycline-transactivator); ApoB: control. (F) Transgene mRNA levels were similar among the entire rTg4510 cohort. (G) Replicate of (F) using a different set of house-keeping gene. Horizontal lines in graphs indicate group mean. Red squares indicate mice with low tau levels.

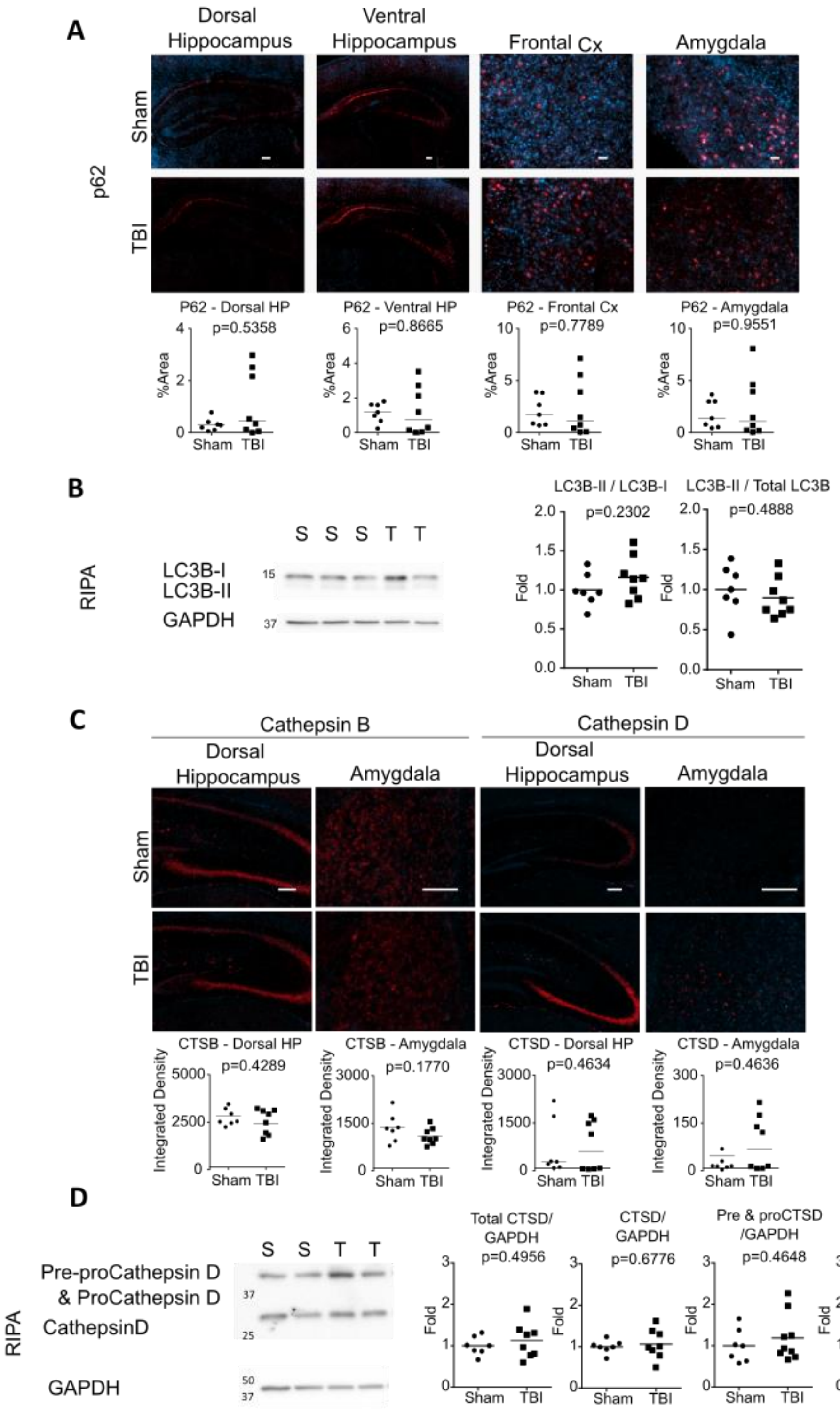

Figure S3. Interfaced TBI at 4.0J did not change p62 accumulation, autophagy initiation, or levels of cathepsin D and B. (A) Immunohistochemistry of p62 was performed to stain autphagosomes. (B) Western blotting and quantification of RIPA brain homogenates using antibodies against LC3B, an marker for autophagy inhibition. (C) Immunohistochemistry of cathepsin B and cathepsin D was performed to stain lysosomes. (D) Western blotting of Pre- & pro-cathepsin D and mature cathepsin D was performed in RIPA brain lysates. Results of immunohistochemistry are quantified in the graphs below the images. Scale bar = 100 µm. Mann-Whitney U tests were used for all p62 staining, CTSD-dHP, and CTSD-Amyg, where horizontal lines in graphs indicate group median. T-tests were used for all analyses, where horizontal lines in graphs indicate group mean.

A

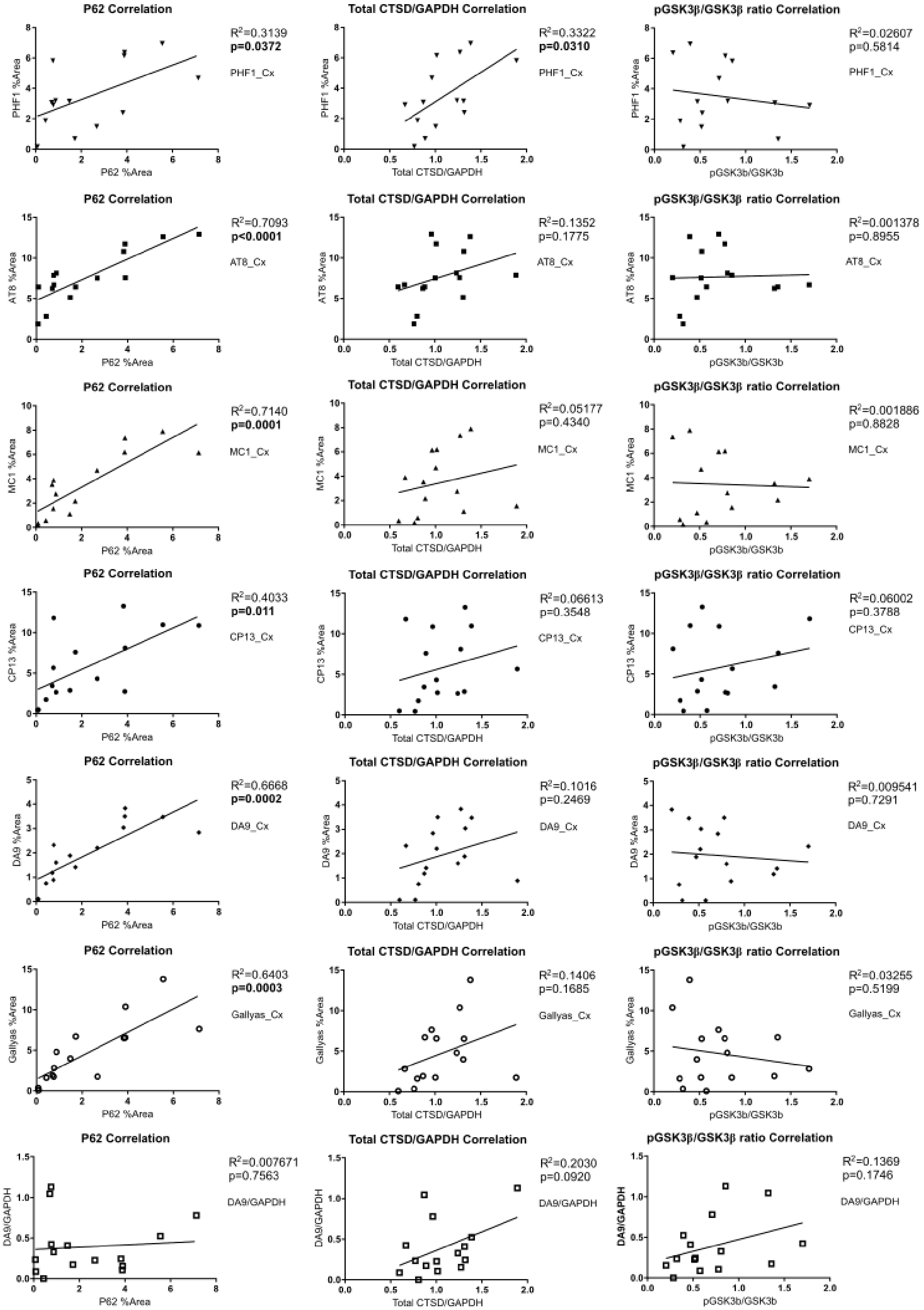
P62 Correlation
PHF1 %Area
P62 %Area
R2=0.3139
p=0.0372
PHF1_Cx
Total CTSD/GAPDH Correlation
Total CTSD/GAPDH
R2=0.3322
p=0.0310
PHF1_Cx
pGSK3β/GSK3β ratio Correlation
pGSK3b/GSK3b
R2=0.02607
p=0.5814
PHF1_Cx
P62 Correlation
AT8 %Area
P62 %Area
R2=0.7093
p<0.0001
AT8_Cx
Total CTSD/GAPDH Correlation
R2=0.1352
p=0.1775
AT8_Cx
pGSK3β/GSK3β ratio Correlation
R2=0.001378
p=0.8955
AT8_Cx
P62 Correlation
MC1 %Area
R2=0.7140
p=0.0001
MC1_Cx
Total CTSD/GAPDH Correlation
R2=0.05177
p=0.4340
MC1_Cx
pGSK3β/GSK3β ratio Correlation
R2=0.001886
p=0.8828
MC1_Cx
P62 Correlation
CP13 %Area
R2=0.4033
p=0.011
CP13_Cx
Total CTSD/GAPDH Correlation
R2=0.06613
p=0.3548
CP13_Cx
pGSK3β/GSK3β ratio Correlation
R2=0.06002
p=0.3788
CP13_Cx
P62 Correlation
DA9 %Area
R2=0.6668
p=0.0002
DA9_Cx
Total CTSD/GAPDH Correlation
R2=0.1016
p=0.2469
DA9_Cx
pGSK3β/GSK3β ratio Correlation
R2=0.009541
p=0.7291
DA9_Cx
P62 Correlation
Gallyas %Area
R2=0.6403
p=0.0003
Gallyas_Cx
Total CTSD/GAPDH Correlation
R2=0.1406
p=0.1685
Gallyas_Cx
pGSK3β/GSK3β ratio Correlation
R2=0.03255
p=0.5199
Gallyas_Cx
P62 Correlation
DA9/GAPDH
R2=0.007671
p=0.7563
DA9/GAPDH
Total CTSD/GAPDH Correlation
R2=0.2030
p=0.0920
DA9/GAPDH
pGSK3β/GSK3β ratio Correlation
R2=0.1369
p=0.1746
DA9/GAPDH

B

	PHF1	AT8	MC1	CP13	DA9	Gallyas	DA9/ GAPDH
p62							
R^2	0.3139	0.7093	0.7140	0.4033	0.6668	0.6403	0.007671
p-value	**0.0372**	**< 0.0001**	**0.0001**	**0.0110**	**0.0002**	**0.0003**	0.7563
CTSD/GAPDH							
R^2	0.3322	0.1352	0.05177	0.06613	0.1016	0.1406	0.2030
p-value	**0.0310**	0.1775	0.4340	0.3548	0.2469	0.1685	0.0920
pGSK3β/GSK3β							
R^2	0.02607	0.001378	0.001886	0.06002	0.009541	0.03255	0.1369
p-value	0.5814	0.8955	0.8828	0.3788	0.7291	0.5199	0.1746

Figure S4. Correlational analyses of tau with autophagolysosomal markers and tau kinase. (A) Plots of Pearson correlation between cortical tau (IHC: PHF1, AT8, CP13, DA9, Gallyas) and tau WB (DA9) with p62, CTSD/GAPDH ratio, and pGSK3b/GSK3b ratio. (B) A summary table of the correlation analyses is shown.

A

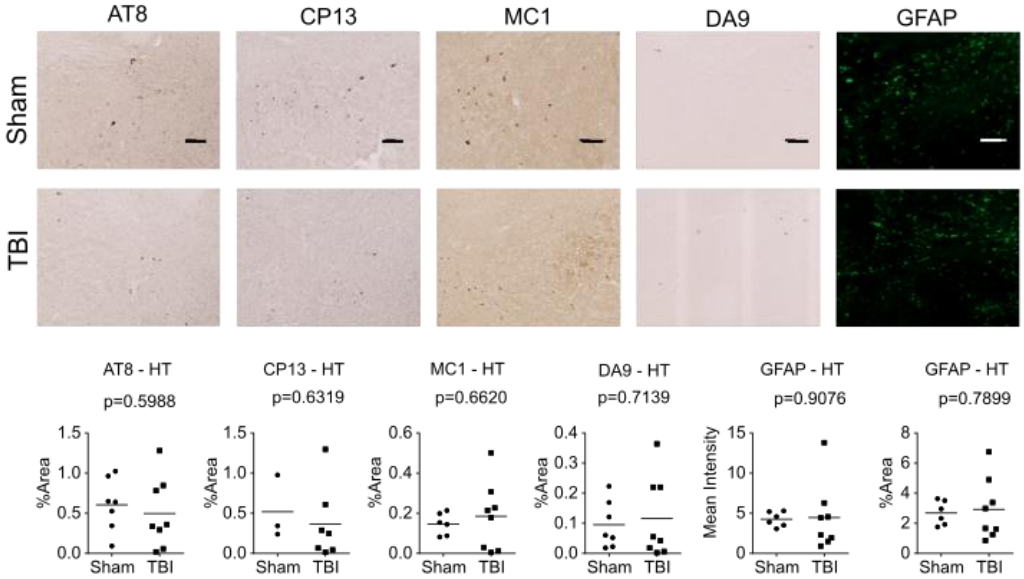

B

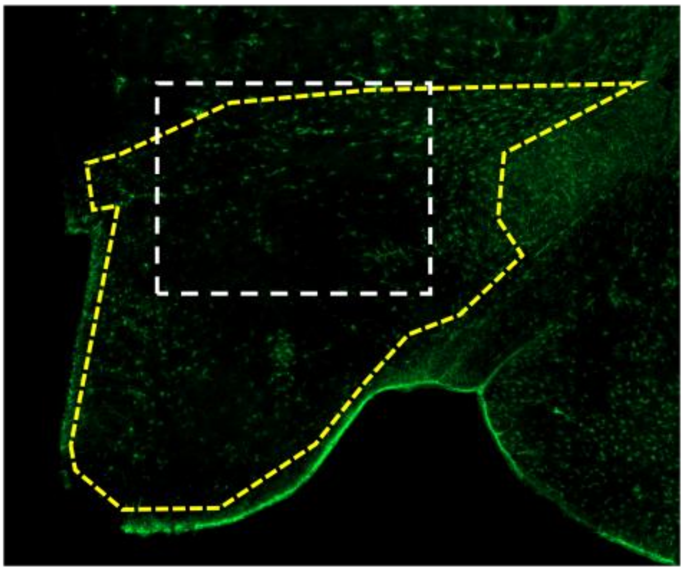

Figure S5. Interfaced TBI did not change tau and astrocyte immunohistochemistry at hypothalamus in rTg4510 mice. (A) Immunohistochemistry of tau (AT8, CP13, MC1, DA9) and astrocyte (GFAP) at hypothalamus were shown. Results are quantified in the graphs below the images. Scale bar = 100 um. T-tests were used for all analyses. Horizontal lines in graphs indicate group mean. (B) An example of hypothalamus quantification is shown, using the GFAP-TBI image. The entire hypothalamus region was selected and used for analyses (yellow dotted polygon), and a subset zoomed-in area (white dotted rectangle) is shown in (A).
